# Pattern of MUC6 expression across 119 different tumor types: A tissue microarray study on 15 412 tumors

**DOI:** 10.1111/pin.13322

**Published:** 2023-04-14

**Authors:** Sebastian Dwertmann Rico, Sebastian J. A. Schliesser, Natalia Gorbokon, David Dum, Anne Menz, Franziska Büscheck, Andrea Hinsch, Maximilian Lennartz, Clara von Bargen, Ahmed A. Bawahab, Andreas M. Luebke, Claudia Hube‐Magg, Christoph Fraune, Patrick Lebok, Till S. Clauditz, Frank Jacobsen, Guido Sauter, Ria Uhlig, Stefan Steurer, Sarah Minner, Andreas H. Marx, Ronald Simon, Eike Burandt, Doris Hoeflmayer, Till Krech, Christian Bernreuther

**Affiliations:** ^1^ Institute of Pathology University Medical Center Hamburg‐Eppendorf Hamburg Germany; ^2^ Department of Pathology, Faculty of Medicine University of Jeddah Jeddah Saudi Arabia; ^3^ Department of Pathology Academic Hospital Fuerth Fuerth Germany; ^4^ Institute of Pathology, Clinical Center Osnabrueck Osnabrueck Germany

**Keywords:** diagnostic, human cancer, immunohistochemistry, MUC6, tissue microarray

## Abstract

Mucin 6 (MUC6) is a secreted gel‐forming mucin covering the surfaces of gastrointestinal and other tissues. Published work demonstrates that MUC6 can also be expressed in several cancer types and can aid in the distinction of different tumor entities. To systematically analyze MUC6 expression in normal and cancerous tissues, a tissue microarray containing 15 412 samples from 119 different tumor types and subtypes as well as 608 samples of 76 different normal tissue types was analyzed by immunohistochemistry. At least a weak MUC6 positivity was seen in 50 of 119 (42%) tumor entities. Thirty‐three tumor entities included tumors with strong positivity. MUC6 immunostaining was most frequent in mucinous carcinomas of the breast (44%), adenocarcinomas of the stomach (30%–40%) and esophagus (35%), and neuroendocrine carcinomas of the colon. Strong MUC6 staining was linked to advanced pT stage (*p* = 0.0464), defective mismatch repair status and right‐sided tumor location (*p* < 0.0001 each) in colorectal cancer, as well as to high tumor grade (*p* = 0.0291), nodal metastasis (*p* = 0.0485), erb‐b2 receptor tyrosine kinase 2 positivity (*p* < 0.0001) and negative estrogen receptor (*p* = 0.0332)/progesterone receptor (*p* = 0.0257) status in breast carcinomas of no special type. The broad range of tumor types with MUC6 expression limits the utility of MUC6 immunohistochemistry for the distinction of different tumor types.

**Abbreviations**
ER: Estrogen receptor
HER2: erb‐b2 receptor tyrosine kinase 2
IHC: Immunohistochemistry
IWGAV: International working group for antibody validation
MMR: Mismatch repair
MUC6: Mucin 6
MSI: Microsatellite instability
PR: Progesterone receptor
RAS: Rat sarcoma virus
TMA: Tissue Microarray
TSA: Traditional serrated adenoma

## INTRODUCTION

Mucin 6 (MUC6) is one of five secreted gel‐forming mucins (MUC2, MUC5AC, MUC5B, MUC6, and MUC19) which are expressed from a gene cluster at chromosome 11p15. Their characteristic cysteine‐rich regions enable oligomerization and formation of the mucin layer which protects the epithelial surfaces from chemical and mechanical stress and microbial pathogens. MUC6 is normally expressed by mucus‐forming cells in the corpus and by the pyloric glands in the antrum of the stomach, by Brunner glands in the duodenum, the gallbladder, parts of the pancreas, and in male and female reproductive organs.^1^ Abnormal MUC6 expression has also been reported from a variety of cancers, and there is growing evidence for a role in tumor development and progression.^2^, ^3^, ^4^, ^5^ For example, MUC6 alterations have been linked to tumor aggressiveness in cancers of the ovaries,^6^ breast,^7^ stomach,^8^ esophagus,^9^ colon,^10^ pancreas,^11^ and bile ducts.^12^, ^13^

In routine pathology, MUC6 immunohistochemistry (IHC) can offer additional diagnostic information for the distinction between gastric and intestinal metaplasia of the Barrett esophagus,^14^ or between intraductal papillary‐mucinous, intraductal tubulo‐papillary and intraductal oncocytic‐papillary neoplasias of the pancreas.^15^ Published studies on MUC6 expression in cancers have reported highly variable data. For example, immunohistochemical MUC6 positivity ranges from 7% to 85% in esophageal adenocarcinoma,^16^, ^17^ from 4% to 93% in cholangiocellular carcinoma,^18^, ^19^ from 24% to 64% in pancreatic ductal adenocarcinoma,^11^, ^20^ from 20% to 100% in cervical adenocarcinoma,^21^, ^22^ and from 0% to 92% in lobular breast carcinoma.^23^, ^24^ These discrepant data are likely due to the use of different antibodies and staining protocols and make it impossible to assess the potential diagnostic significance of MUC6 for the distinction of tumor entities.

This study was designed to provide a comprehensive analysis of MUC6 expression across a broad range of different tumor entities. For this purpose, >15 000 tissue samples from 119 different tumor types and subtypes, and 76 non‐neoplastic tissues were evaluated by IHC in a tissue microarray (TMA) format under highly standardized conditions.

## MATERIAL AND METHODS

### TMAs

TMAs composed of normal and tumorous tissues were employed for this study. The normal tissue TMA contained eight samples from eight different donors from each of 76 different normal tissue types. Normal tissues were obtained from donors who underwent surgery for reasons other than cancer. The cancer TMAs contained a total of 15 412 primary tumors from 119 tumor types and subtypes. Histopathological data including grade, pT and pN status was available from 524 ovarian cancers, 259 endometrial cancers, 598 pancreatic cancers, 327 gastric cancers, and 1475 breast cancers. The breast cancer dataset also included molecular information on estrogen receptor (ER), progesterone receptor (PR), erb‐b2 receptor tyrosine kinase 2 (HER2) as well as follow‐up information on a subset of 877 patients with a median follow‐up time of 49 months (range, 1–88). The colon cancer TMA included data on lymphatic infiltration (L), mismatch repair (MMR) protein status, and rat sarcoma virus (RAS) mutation status. The composition of both normal and cancer TMAs is described in detail in the results section. All samples were from the archives of the Institutes of Pathology, University Medical Center Hamburg‐Eppendorf, Germany, the Institute of Pathology, Clinical Center Osnabrueck, Germany, and Department of Pathology, Academic Hospital Fuerth, Germany. Tissues were fixed in 4% buffered formalin (10% dilution of saturated [38%] formalin solution) and then embedded in paraffin. The TMA manufacturing process was described earlier in detail.^25^, ^26^ TMA tissue spot diameter was 0.6 mm. The use of archived remnants of diagnostic tissues for manufacturing of TMAs and their analysis for research purposes as well as patient data analysis has been approved by local laws (HmbKHG, §12) and by the local ethics committee (Ethics Commission Hamburg, WF‐049/09). All work has been carried out in compliance with the Helsinki Declaration.

### IHC

Freshly cut TMA sections were immunostained on one day and in one experiment. Slides were deparaffinized and exposed to heat‐induced antigen retrieval for 5 min in an autoclave at 121°C in pH 7.8 buffer. Primary antibody (MSVA‐806R, rabbit recombinant, MS Validated Antibodies) was used for MUC6 detection. The antibody was applied at 37°C for 60 min at a dilution of 1:150. For the purpose of antibody validation, the normal TMA was also analyzed with the anti‐MUC6 mouse monoclonal antibody CLH5 (Bio SB, cat. #BSB6171, ready‐to‐use) after antigen retrieval at pH 9 (Agilent/Dako Omnis Target Retrieval Solution High pH, cat. #K8004). Bound antibody was then visualized using the EnVision Flex reagent (Agilent #52023) according to the manufacturer's directions. For tumor tissues, the percentage of positive neoplastic cells was estimated, and the staining intensity was semiquantitatively recorded (0, 1+, 2+, 3+). For statistical analyses, the staining results were categorized into four groups. Tumors without any staining were considered negative. Tumors with 1+ staining intensity in ≤70% of tumor cells or 2+ intensity in ≤30% of tumor cells were considered weakly positive. Tumors with 1+ staining intensity in >70% of tumor cells, 2+ intensity in 31%–70%, or 3+ intensity in ≤30% of tumor cells were considered moderately positive. Tumors with 2+ intensity in >70% or 3+ intensity in >30% of tumor cells were considered strongly positive.

### Statistics

Statistical calculations were performed with JMP 16 software (SAS Institute Inc.). Contingency tables and the χ^2^ test were performed to search for associations between MUC6 immunostaining and tumor phenotype. Survival curves were calculated according to Kaplan‐Meier. The log‐rank test was applied to detect significant differences between groups. A *p*‐value of ≤0.05 was considered as statistically significant.

## RESULTS

### Technical issues

A total of 11 685 (75.8%) of 15 412 tumor samples were interpretable in our TMA analysis. The remaining 3727 (24.2%) samples were not interpretable due to the lack of unequivocal tumor cells or a lack of the entire tissue spot. On the normal tissue TMA, enough samples (≥4) were always analyzable per tissue type to determine MUC6 staining patterns in individual cell types.

### MUC6 in normal tissue

**Figure 1:**
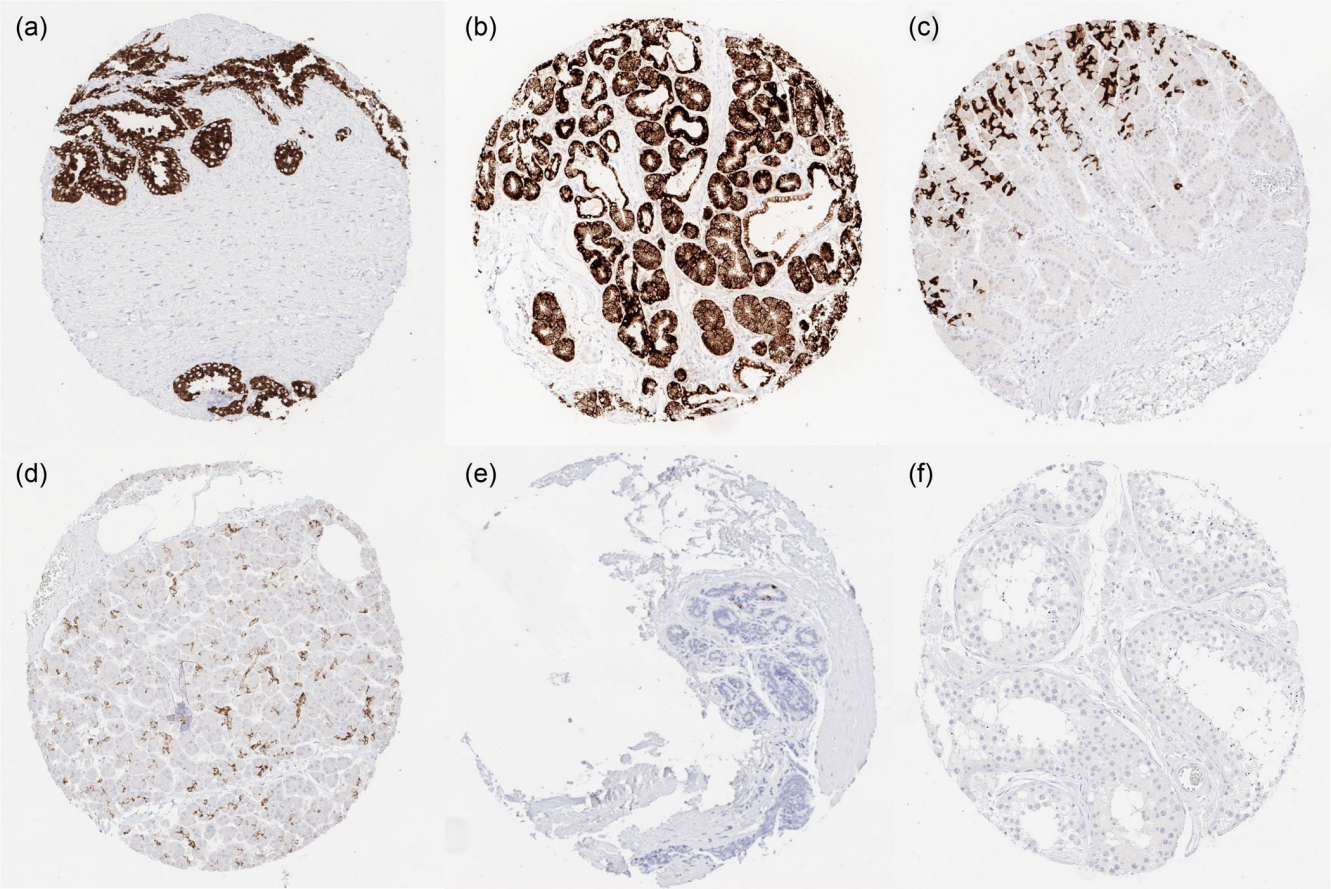
Mucin 6 (MUC6) immunostaining in normal tissue. The panels show a diffuse and intense MUC6 staining in epithelial cells of (a) seminal vesicles and (b) in Brunner glands of the duodenum, (c) a strong MUC6 staining of mucous secreting glands of the stomach, (d) a moderate to strong staining of intercalated ducts in the pancreas, (e) a focal MUC6 staining of some luminal cells of breast glands, and (f) a complete absence of staining in the testis.

Strongest cytoplasmic MUC6 staining was seen in epithelial cells of seminal vesicles, Brunner glands of the duodenum, and the mucous secreting glands of the stomach. Positive staining was also consistently found in intercalated and interlobular ducts of the pancreas, in small juxtaportal bile ducts (but not in the portal bile ducts), in gallbladder surface epithelium (not in all samples), in the cauda (more intense) and caput (less intense and more focal) of the epididymis, in scattered cells of the fallopian tube, and (weakly) in endocervical glands. A few scattered MUC6 positive cells were also seen at least in some samples of collecting ducts of the kidney, breast glands, endometrium in pregnancy, and in the trophoblast of the first trimenon placenta. Representative images are shown in Figure 1. All these findings were observed by both MSVA‐806R and CLH5 antibodies (Supporting Information: Supplementary Figure 1). Normal tissues without detectable MUC6 staining included skin and nonkeratinizing squamous epithelium of oral cavity, lip, tonsil surface and crypts, ectocervix, esophagus, stomach surface epithelium, colon mucosa, corpuscles of Hassall's in the thymus, mesenchymal tissues (myometrium, lymphatic and hematopoietic cells), hepatocytes, proximal and distal tubuli as well as glomeruli of the kidney, salivary glands, prostate, testis, respiratory epithelium, lung, ovary including corpus luteum and follicular cysts, mature placenta, adrenal gland, thyroid, cerebrum, cerebellum, adeno‐ and neurohypophysis.

### MUC6 in neoplastic tissues

**Figure 2:**
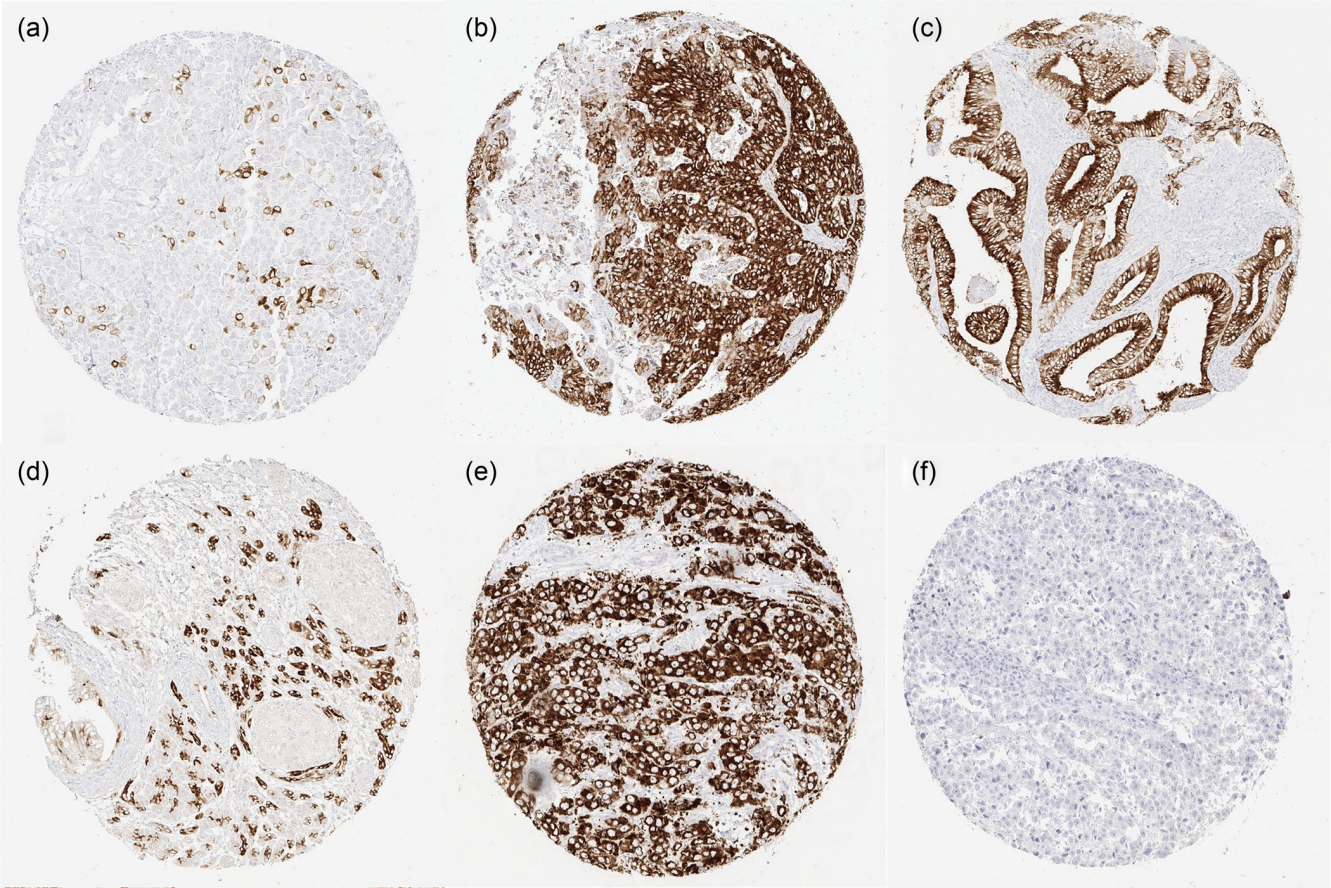
Mucin 6 (MUC6) immunostaining in cancer. The panels show (a) a scattered MUC6 staining in muscle‐invasive urothelial carcinoma of the urinary bladder, (b) strong staining in gastric adenocarcinoma, (c) endometrioid endometrial carcinoma, (d) ductal adenocarcinoma of the pancreas, (e) recurrent adenocarcinoma of the prostate, and (f) absence of staining in seminoma.

**Figure 3:**
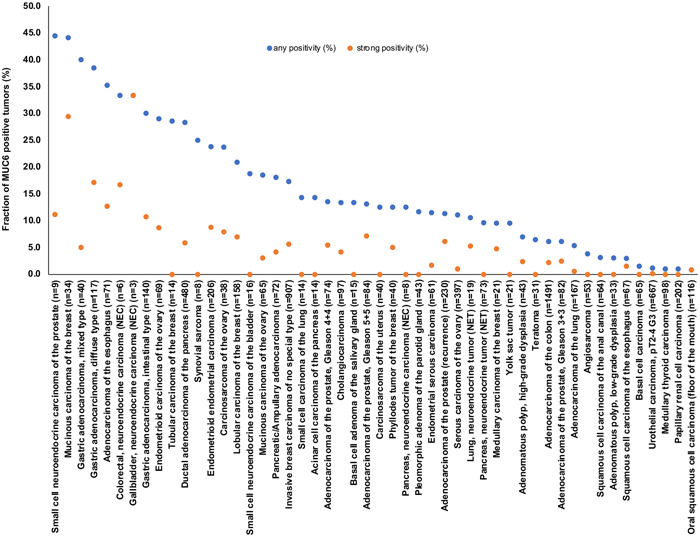
Ranking order of mucin 6 (MUC6) immunostaining in tumors. Both the frequency of positive cases (blue dots) and the frequency of strongly positive cases (orange dots) are shown.

MUC6 staining was always cytoplasmic in tumor tissues. Representative pictures of MUC6 positive and negative cancers are shown in Figure 2. Positive MUC6 immunostaining was detectable in 864 (7.4%) of the 11 685 analyzable tumors, including 387 (3.3%) with weak, 220 (1.9%) with moderate, and 257 (2.2%) with strong positivity. Overall, 50 (42%) of 119 tumor categories showed a detectable MUC6 staining of at least one tumor with 33 (28%) tumor categories showing at least in one case a strong positivity (Table 1). The tumor categories with the highest rate of positive staining included mucinous carcinomas of the breast (44%), gastric adenocarcinomas (30%–40%), esophageal adenocarcinomas (35%), neuroendocrine carcinomas of the colon and gallbladder (33% each), endometrioid carcinomas of the ovary (29%), tubular carcinomas of the breast (29%), ductal adenocarcinomas of the pancreas (28%), endometrioid endometrial carcinomas (24%), carcinosarcomas of the ovary (24%), and lobular carcinoma of the breast (21%). A graphical representation of a ranking order of MUC6 positive and strongly positive cancers is given in Figure 3. The relationship between MUC6 positivity and histopathological tumor phenotype is shown in Table 2. Strong MUC6 staining was linked to advanced pT stage (*p* = 0.0464), defective MMR status, and right‐sided tumor location (*p* < 0.0001 each) in colorectal cancer, as well as to high tumor grade (*p* = 0.0291), nodal metastasis (*p* = 0.0485), HER2 positivity (*p* < 0.0001) and a negative ER (*p* = 0.0332)/PR (*p* = 0.0257) status in breast carcinomas of no special type. MUC6 expression was unrelated to clinico‐pathological features in serous ovarian carcinomas, endometrioid endometrial carcinomas, ductal adenocarcinomas of the pancreas, and in gastric adenocarcinomas.

**Table 1:** Mucin 6 (MUC6) immunostaining in human tumors.

			MUC6 immunostaining				
	Tumor entity	On tissue microarray (*n*)	Analyzable (*n*)	Negative (%)	Weak (%)	Moderate (%)	Strong (%)
Tumors of the skin	Pilomatricoma	35	29	100.0	0.0	0.0	0.0
	Basal cell carcinoma	88	65	98.5	0.0	1.5	0.0
	Benign nevus	29	23	100.0	0.0	0.0	0.0
	Squamous cell carcinoma of the skin	90	86	100.0	0.0	0.0	0.0
	Malignant melanoma	46	41	100.0	0.0	0.0	0.0
	Merkel cell carcinoma	46	31	100.0	0.0	0.0	0.0
Tumors of the head and neck	Squamous cell carcinoma of the larynx	109	83	100.0	0.0	0.0	0.0
	Squamous cell carcinoma of the pharynx	60	51	100.0	0.0	0.0	0.0
	Oral squamous cell carcinoma (floor of the mouth)	130	116	99.1	0.0	0.0	0.9
	Pleomorphic adenoma of the parotid gland	50	43	88.4	2.3	9.3	0.0
	Warthin tumor of the parotid gland	49	45	100.0	0.0	0.0	0.0
	Basal cell adenoma of the salivary gland	15	15	86.7	13.3	0.0	0.0
Tumors of the lung, pleura and thymus	Adenocarcinoma of the lung	196	167	94.6	3.0	1.8	0.6
	Squamous cell carcinoma of the lung	80	64	100.0	0.0	0.0	0.0
	Small cell carcinoma of the lung	16	14	85.7	14.3	0.0	0.0
	Mesothelioma, epithelioid	39	17	100.0	0.0	0.0	0.0
	Mesothelioma, biphasic	76	44	100.0	0.0	0.0	0.0
	Thymoma	29	25	100.0	0.0	0.0	0.0
	Lung, neuroendocrine tumor (NET)	19	19	89.5	5.3	0.0	5.3
Tumors of the female genital tract	Squamous cell carcinoma of the vagina	78	50	100.0	0.0	0.0	0.0
	Squamous cell carcinoma of the vulva	130	102	100.0	0.0	0.0	0.0
	Squamous cell carcinoma of the cervix	128	100	100.0	0.0	0.0	0.0
	Endometrioid carcinoma of the uterine corpus	236	206	76.2	8.3	6.8	8.7
	Endometrial serous carcinoma	82	61	88.5	6.6	3.3	1.6
	Carcinosarcoma of the uterus	48	40	87.5	7.5	5.0	0.0
	Endometrial carcinoma, high‐grade, G3	13	13	100.0	0.0	0.0	0.0
	Endometrial clear cell carcinoma	8	7	100.0	0.0	0.0	0.0
	Endometrioid carcinoma of the ovary	110	69	71.0	15.9	4.3	8.7
	Serous carcinoma of the ovary	559	397	88.9	8.1	2.0	1.0
	Mucinous carcinoma of the ovary	96	65	81.5	6.2	9.2	3.1
	Clear cell carcinoma of the ovary	50	34	100.0	0.0	0.0	0.0
	Carcinosarcoma of the ovary	47	38	76.3	10.5	5.3	7.9
	Brenner tumor	9	8	100.0	0.0	0.0	0.0
Tumors of the breast	Invasive breast carcinoma of no special type	1345	907	82.7	8.3	3.4	5.6
	Lobular carcinoma of the breast	293	158	79.1	10.1	3.8	7.0
	Medullary carcinoma of the breast	26	21	90.5	4.8	0.0	4.8
	Tubular carcinoma of the breast	27	14	71.4	28.6	0.0	0.0
	Mucinous carcinoma of the breast	58	34	55.9	2.9	11.8	29.4
	Phyllodes tumor of the breast	50	40	87.5	7.5	0.0	5.0
Tumors of the digestive system	Adenomatous polyp, low‐grade dysplasia	50	33	97.0	3.0	0.0	0.0
	Adenomatous polyp, high‐grade dysplasia	50	43	93.0	4.7	0.0	2.3
	Adenocarcinoma of the colon	1882	1491	93.9	2.8	1.1	2.1
	Gastric adenocarcinoma, diffuse type	176	117	61.5	12.0	9.4	17.1
	Gastric adenocarcinoma, intestinal type	174	140	70.0	9.3	10.0	10.7
	Gastric adenocarcinoma, mixed type	62	40	60.0	15.0	20.0	5.0
	Adenocarcinoma of the esophagus	83	71	64.8	5.6	16.9	12.7
	Squamous cell carcinoma of the esophagus	76	67	97.0	0.0	1.5	1.5
	Squamous cell carcinoma of the anal canal	89	64	96.9	0.0	3.1	0.0
	Cholangiocarcinoma	113	97	86.6	7.2	2.1	4.1
	Hepatocellular carcinoma	50	45	100.0	0.0	0.0	0.0
	Ductal adenocarcinoma of the pancreas	612	480	71.7	14.6	7.9	5.8
	Pancreatic/ampullary adenocarcinoma	89	72	81.9	5.6	8.3	4.2
	Acinar cell carcinoma of the pancreas	16	14	85.7	0.0	14.3	0.0
	Gastrointestinal stromal tumor	50	44	100.0	0.0	0.0	0.0
	Appendix, NET	22	11	100.0	0.0	0.0	0.0
	Colorectal, NET	12	11	100.0	0.0	0.0	0.0
	Ileum, NET	49	36	100.0	0.0	0.0	0.0
	Pancreas, NET	97	73	90.4	4.1	5.5	0.0
	Colorectal, neuroendocrine carcinoma (NEC)	12	6	66.7	0.0	16.7	16.7
	Gallbladder, NEC	4	3	66.7	0.0	0.0	33.3
	Pancreas, NEC	14	8	87.5	0.0	12.5	0.0
Tumors of the urinary system	Non‐invasive papillary urothelial carcinoma, pTa G2 low‐grade	177	117	100.0	0.0	0.0	0.0
	Non‐invasive papillary urothelial carcinoma, pTa G2 high‐grade	141	82	100.0	0.0	0.0	0.0
	Non‐invasive papillary urothelial carcinoma, pTa G3	187	78	100.0	0.0	0.0	0.0
	Urothelial carcinoma, pT2‐4 G3	1206	667	98.8	0.9	0.1	0.1
	Small cell neuroendocrine carcinoma of the bladder	20	16	81.3	18.8	0.0	0.0
	Clear cell renal cell carcinoma	857	719	100.0	0.0	0.0	0.0
	Papillary renal cell carcinoma	255	202	99.0	1.0	0.0	0.0
	Clear cell (tubulo) papillary renal cell carcinoma	21	17	100.0	0.0	0.0	0.0
	Chromophobe renal cell carcinoma	131	107	100.0	0.0	0.0	0.0
	Oncocytoma	177	134	100.0	0.0	0.0	0.0
Tumors of the male genital organs	Adenocarcinoma of the prostate, Gleason 3 + 3	83	82	93.9	2.4	1.2	2.4
	Adenocarcinoma of the prostate, Gleason 4 + 4	80	74	86.5	5.4	2.7	5.4
	Adenocarcinoma of the prostate, Gleason 5 + 5	85	84	86.9	4.8	1.2	7.1
	Adenocarcinoma of the prostate (recurrence)	258	230	88.7	3.0	2.2	6.1
	Small cell neuroendocrine carcinoma of the prostate	19	9	55.6	22.2	11.1	11.1
	Seminoma	621	566	100.0	0.0	0.0	0.0
	Embryonal carcinoma of the testis	50	19	100.0	0.0	0.0	0.0
	Yolk sac tumor	50	21	90.5	4.8	4.8	0.0
	Teratoma	50	31	93.5	6.5	0.0	0.0
	Squamous cell carcinoma of the penis	80	74	100.0	0.0	0.0	0.0
Tumors of endocrine organs	Adenoma of the thyroid gland	113	107	100.0	0.0	0.0	0.0
	Papillary thyroid carcinoma	391	362	100.0	0.0	0.0	0.0
	Follicular thyroid carcinoma	154	141	100.0	0.0	0.0	0.0
	Medullary thyroid carcinoma	111	98	99.0	1.0	0.0	0.0
	Anaplastic thyroid carcinoma	45	39	100.0	0.0	0.0	0.0
	Adrenal cortical adenoma	50	25	100.0	0.0	0.0	0.0
	Adrenal cortical carcinoma	26	21	100.0	0.0	0.0	0.0
	Phaeochromocytoma	50	42	100.0	0.0	0.0	0.0
Tumors of hemotopoetic and lymphoid tissues	Hodgkin lymphoma	103	87	100.0	0.0	0.0	0.0
	Small lymphocytic lymphoma, B‐cell type (B‐SLL/B‐CLL)	50	47	100.0	0.0	0.0	0.0
	Diffuse large B‐cell lymphoma (DLBCL)	113	108	100.0	0.0	0.0	0.0
	Follicular lymphoma	88	83	100.0	0.0	0.0	0.0
	T‐cell non‐Hodgkin lymphoma	25	22	100.0	0.0	0.0	0.0
	Mantle cell lymphoma	18	18	100.0	0.0	0.0	0.0
	Marginal zone lymphoma	16	11	100.0	0.0	0.0	0.0
	DLBCL in the testis	16	16	100.0	0.0	0.0	0.0
	Burkitt lymphoma	5	3	100.0	0.0	0.0	0.0
Tumors of soft tissue and bone	Tendosynovial giant cell tumor	45	33	100.0	0.0	0.0	0.0
	Granular cell tumor	53	20	100.0	0.0	0.0	0.0
	Leiomyoma	50	47	100.0	0.0	0.0	0.0
	Leiomyosarcoma	87	79	100.0	0.0	0.0	0.0
	Liposarcoma	132	88	100.0	0.0	0.0	0.0
	Malignant peripheral nerve sheath tumor	13	11	100.0	0.0	0.0	0.0
	Myofibrosarcoma	26	23	100.0	0.0	0.0	0.0
	Angiosarcoma	73	53	96.2	0.0	3.8	0.0
	Angiomyolipoma	91	51	100.0	0.0	0.0	0.0
	Dermatofibrosarcoma protuberans	21	12	100.0	0.0	0.0	0.0
	Ganglioneuroma	14	13	100.0	0.0	0.0	0.0
	Kaposi sarcoma	8	2	100.0	0.0	0.0	0.0
	Neurofibroma	117	105	100.0	0.0	0.0	0.0
	Sarcoma, not otherwise specified	74	64	100.0	0.0	0.0	0.0
	Paraganglioma	41	41	100.0	0.0	0.0	0.0
	Ewing sarcoma	23	10	100.0	0.0	0.0	0.0
	Rhabdomyosarcoma	6	3	100.0	0.0	0.0	0.0
	Schwannoma	121	116	100.0	0.0	0.0	0.0
	Synovial sarcoma	12	8	75.0	12.5	12.5	0.0
	Osteosarcoma	43	25	100.0	0.0	0.0	0.0
	Chondrosarcoma	38	14	100.0	0.0	0.0	0.0

**Table 2:** Mucin 6 (MUC6) immunostaining and tumor phenotype in breast cancers of no special type, colon adenocarcinomas, endometrioid endometrium carcinoma, serous ovarian cancers, pancreatic adenocarcinomas, and gastric cancers.

				MUC6 immunohistochemistry result				
			*n*	Negative (%)	Weak (%)	Moderate (%)	Strong (%)	*p*
Breast cancer of no special type	Tumor stage	pT1	428	84.6	7.5	2.8	5.1	0.9324
		pT2	322	82.3	8.4	3.7	5.6	
		pT3‐4	76	80.3	9.2	5.3	5.3	
	Grade	G1	138	88.4	9.4	0.7	1.4	0.0291
		G2	404	80.9	8.9	4.2	5.9	
		G3	314	84.4	6.1	3.8	5.7	
	Nodal stage	pN0	335	86.9	6.3	3.0	3.9	0.0485
		pN+	273	78.4	8.9	4.4	7.3	
	erb‐b2 receptor tyrosine kinase 2 (HER2) status	Negative	684	86.3	7.5	2.6	3.7	<0.0001
		Positive	102	64.7	11.8	6.9	16.7	
	Estrogen receptor (ER) status	Negative	159	78.0	9.4	2.5	10.1	0.0332
		Positive	594	84.5	7.9	3.5	4.0	
	Progesterone receptor (PR) status	Negative	309	79.3	10.7	2.9	7.1	0.0257
		Positive	468	85.7	7.1	3.8	3.4	
	Triple negative	No	625	82.6	8.5	3.7	5.3	0.1740
		Yes	106	89.6	6.6	0.9	2.8	
Colon adenocarcinoma	Tumor stage	pT1	52	98.1	0.0	0.0	1.9	0.0464
		pT2	293	93.9	1.4	2.0	2.7	
		pT3	790	93.5	3.4	0.6	2.4	
		pT4	294	94.2	3.7	1.4	0.7	
	Nodal stage	pN0	744	93.7	2.4	1.2	2.7	0.2233
		pN+	672	94.0	3.6	0.9	1.5	
	Lymph vessel stage	L0	552	94.4	2.2	1.3	2.2	0.5167
		L1	838	93.4	3.5	1.0	2.1	
	Tumor localization	Left colon	1064	96.2	1.9	0.7	1.2	<0.0001
		Right colon	370	87.0	5.9	2.4	4.6	
	Mismatch repair (MMR) status	Defective	74	73.0	6.8	2.7	17.6	<0.0001
		Proficient	1042	95.9	2.3	0.7	1.2	
	Rat sarcoma virus (RAS) mutation status	Mutated	316	97.5	1.9	0.3	0.3	0.161
		Wildtype	405	95.1	2.2	1.0	1.7	
	BRAF mutation status	Mutated	18	72.2	11.1	11.1	5.6	0.0497
		Wildtype	112	92.9	4.5	2.7	0.0	
Endometrioid endometrial cancer	Tumor stage	pT1	107	77.6	8.4	4.7	9.3	0.896
		pT2	22	81.8	4.5	9.1	4.5	
		pT3‐4	33	75.8	6.1	9.1	9.1	
	Nodal stage	pN0	48	77.1	6.3	14.6	2.1	0.0549
		pN+	27	85.2	0.0	3.7	11.1	
Serous ovarian cancer	Tumor stage	pT1	26	88.5	11.5	0.0	0.0	0.6084
		pT2	36	94.4	5.6	0.0	0.0	
		pT3	203	88.7	7.9	2.5	1.0	
	Nodal stage	pN0	69	92.8	4.3	1.4	1.4	0.8596
		pN1	129	90.7	7.0	1.6	0.8	
Pancreatic adenocarcinoma	Tumor stage	pT1	12	66.7	16.7	0	16.7	0.6002
		pT2	60	73.3	13.3	10	3.3	
		pT3	334	72.8	13.5	7.8	6	
		pT4	26	57.7	23.1	11.5	7.7	
	Grade	1	16	62.5	12.5	6.3	18.8	0.2585
		2	304	70.7	15.8	8.9	4.6	
		3	92	75	9.8	6.5	8.7	
	Nodal stage	pN0	89	75.3	10.1	7.9	6.7	0.6367
		pN+	342	70.8	15.2	8.2	5.8	
Stomach cancer	Laurén type	Diffuse	46	63.0	15.2	13.0	8.7	0.5418
		Intestinal	70	70.0	10.0	8.6	11.4	
		Mixed	40	60.0	15.0	20.0	5.0	
	Tumor stage	pT1‐2	42	64.3	14.3	7.1	14.3	0.3028
		pT3	85	65.9	15.3	14.1	4.7	
		pT4	71	70.4	12.7	5.6	11.3	
	Nodal stage	pN0	49	67.3	14.3	8.2	10.2	0.7641
		pN1	44	63.6	15.9	9.1	11.4	
		pN2	41	80.5	7.3	7.3	4.9	
		pN3	64	60.9	17.2	12.5	9.4	
	MMR status	MMR defective	30	63.3	6.7	10.0	20.0	0.2739
		MMR proficient	173	66.5	15.0	9.2	9.2	

## DISCUSSION

More than 11 600 samples from 119 different tumor types and subtypes were successfully analyzed for MUC6 expression in our study. The analysis identified 51 tumor entities with at least occasional MUC6 expression. Among the 16 tumor entities for which MUC6 expression had not been previously reported there were several clinically relevant tumor types such as for example serous and endometrioid ovarian carcinomas, neuroendocrine tumors of the pancreas, small cell neuroendocrine carcinomas of the prostate and the urinary bladder, as well as testicular yolk sac tumors. Most MUC6 positive cancers showed a glandular differentiation. The highest prevalences of MUC6 expression were seen in carcinomas of the breast, adenocarcinomas of the stomach, esophagus, colorectum, and the pancreas, as well as in carcinomas of the endometrium and the ovary. It is of note that a considerable fraction of these tumor types is derived from epithelial tissues (i.e., colon mucosa, endometrium) that do not regularly express MUC6.

**Figure 4:**
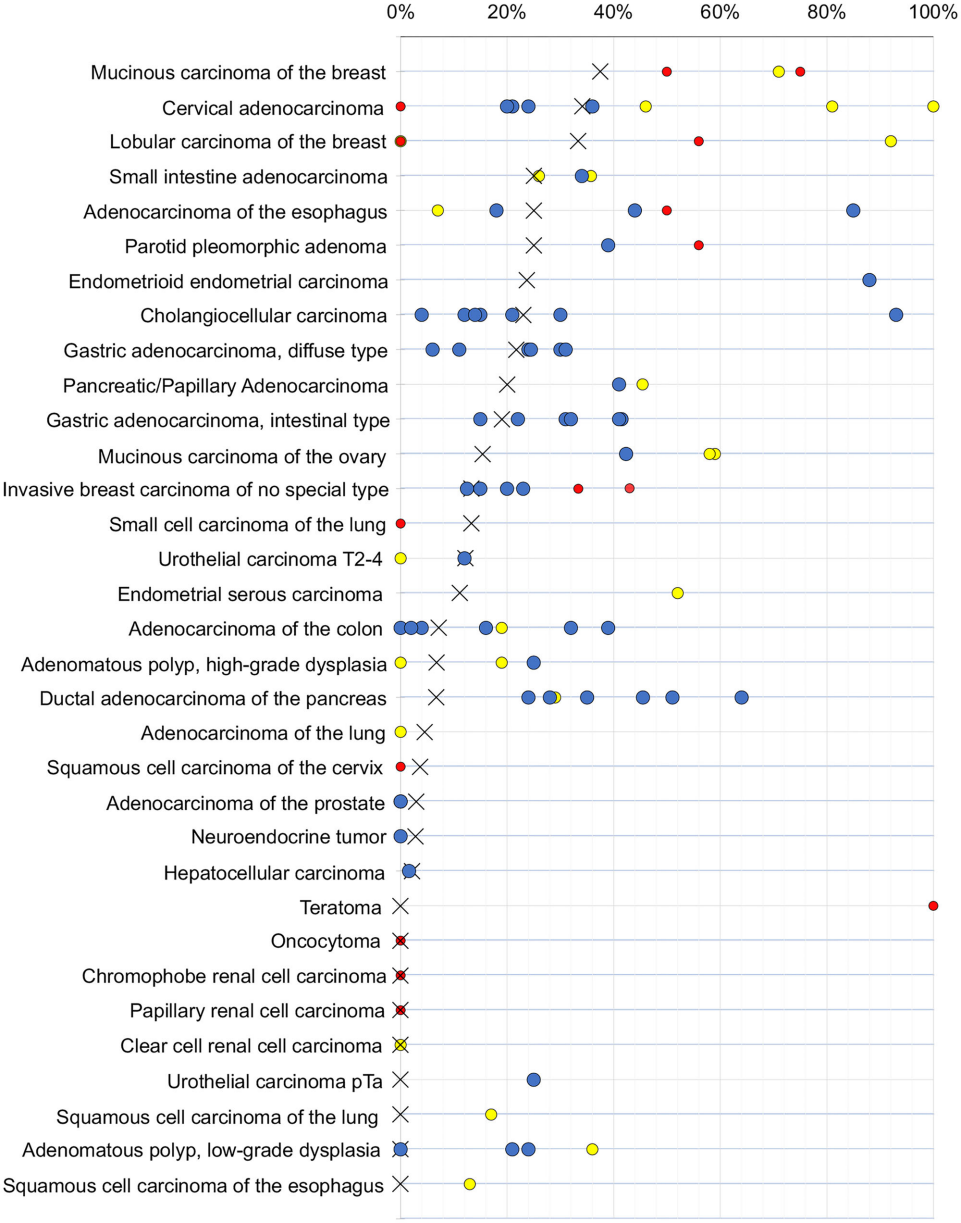
Mucin 6 (MUC6) positivity in the literature. All literature studies used anti‐MUC6 antibodies other than MSVA‐806R. An “X” indicates the fraction of MUC6 positive tumors in the present study, dots indicate the reported frequencies from the literature for comparison: red dots mark studies with ≤10 tumors, yellow dots mark studies with 11–25 tumors, and blue dots mark studies with >25 tumors.

The ranking list of tumors according to their MUC6 positivity rate is an important result of this study. Given the maximal standardization of our experimental procedure and the rigorous validation of our reagents, we assume that this list reflects the relative importance of MUC6 expression for these tumors. Although the absolute positivity rates obtained in our study are specific for our assay, we expect that other protocols or the use of other highly specific antibodies would result in a comparable ranking order. The comparison of our data with previous data collected from the literature (Figure 4) demonstrates that a comparable ranking order could not be generated from existing literature data because of the very high variability of published results. These are reflective of the range of technical issues connected to such studies, including the use of different antibodies, IHC protocols and scoring strategies which all can massively impact the outcome of immunohistochenmical analyses. These observations also highlight the important role of large‐scale studies involving many different tumor categories for assessing the utility of diagnostic immunohistochemical markers.

Our findings do not suggest a major role of MUC6 IHC for the distinction of tumor entities. The fact that none of the analyzed tumor categories showed MUC6 positivity in more than 40% of cases makes it clear that the absence of MUC6 immunostaining cannot exclude any tumor entity. In principle, MUC6 IHC would provide the highest level of diagnostic information with respect to tumor types that are always MUC6 negative. A positive MUC6 immunostaining would literally exclude such tumor entities from diagnostic considerations. In the present analysis, 59 of 119 analyzed tumor types and subtypes did not show MUC6 immunostaining. These for example included all different categories of squamous cell carcinomas, mesotheliomas, and hepatocellular carcinomas. Also, renal cell and thyroidal carcinomas showed MUC6 positivity in <1% of cases and are therefore considered unlikely sources for MUC6 positive metastases.

The existence of sizable subgroups of MUC6 positive and negative tumors within many clinically relevant cancer types raises the question of a possible prognostic or predictive role of MUC6 expression. The case numbers analyzed in this study were large enough to search for associations between MUC6 staining and features of tumor aggressiveness in six different tumor entities. That MUC6 positivity was linked to advanced stage in colorectal adenocarcinoma and to high Bloom‐Richardson‐Elston grade, nodal metastasis and molecular features that are related to aggressive tumor phenotype (ER negative, PR negative, and HER2 positive) in breast cancers of no special type demonstrates that high MUC6 expression can be linked to a more aggressive tumor behavior. As MUC6 is usually not expressed in normal colorectal epithelial cells, our findings suggest that a neo‐expression of MUC6 can parallel tumor progression in at least some tumor types. Of note, Betge et al.^10^ had previously analyzed MUC6 in 381 colorectal adenocarcinomas and found that high MUC6 expression was linked to long progression‐free (*p* = 0.024) and cancer‐specific survival (*p* = 0.043). Other authors had addressed the prognostic role of MUC6 expression in 225 gastric cancers,^8^ 101 pancreatic cancers,^11^ 85–100 cholangiocarcinomas,^12^, ^13^ 73 salivary gland carcinomas,^27^ and 36 ovarian cancers.^6^ Most of these studies had suggested a better prognosis in patients with high MUC6 expression. Considering these controversial results and that we could not find any associations between MUC6 expression and histological features of aggressive tumor behavior in four further tumor entities, we believe that MUC6 expression is not a strong or universal feature of aggressive tumor behavior. That MUC6 expression was tightly linked to microsatellite instability (MSI) in colorectal carcinoma in our study is consistent with two recent studies on traditional and sessile serrated adenomas. Tanaka et al. described complete absence of MUC6 staining in 73 cases of traditional serrated adenoma (TSA), a precursor lesion for microsatellite stable colorectal cancer.^28^ Murakami et al. found a MUC6 positivity in six of seven sessile serrated adenomas, known to represent a precursor lesion for MSI colorectal cancer.^29^

Given the large scale of our study, emphasis was placed on an adequate validation of our assay. According to the recommendations of the international working group for antibody validation (IWGAV), we validated our approach by comparing our IHC findings in normal tissues with data obtained by another independent anti‐MUC6 antibody and RNA data derived from two different publicly accessible databases.^30^, ^31^, ^32^, ^33^ The use of 76 different normal tissues for antibody validation ensured that a very large fraction of the proteins expressed in cells of adult humans were exposed to our antibody. Such a broad tissue validation increases the likelihood for detecting possible cross‐reactivities. Validity of our assay was supported by the detection of MUC6 immunostaining in all organs with unequivocal MUC6 RNA expression (stomach, duodenum, seminal vesicles, pancreas, cervix uteri). The additional MUC6 stains obtained in epithelial cells of the epididymis, juxtaportal bile ducts of the liver, gallbladder epithelium, breast epithelium, endometrium of pregnancy, scattered cells of the fallopian tube, some collecting ducts of the kidney, and placental trophoblastic cells were confirmed by the use of the independent second antibody CLH5. That MUC6 RNA expression had not been described for these organs is probably due to the small number of MUC6 positive cells in these tissues which may not have resulted in the detection of MUC6 RNAs in whole organ analyses because of a too high dilution of MUC6 RNAs if whole organ RNAs were analyzed.

In summary, the results of this study show that MUC6 is expressed in a broad range of different tumor entities. Given that the rate of MUC6 positivity does not exceed 40% in any tumor entitity, MUC6 expression analysis is not suited for the distinction of tumors of different sites of origin. The prognostic and predictive roles of MUC6 expression need to be further assessed.

## AUTHOR CONTRIBUTIONS

Sebastian Dwertmann Rico, Ronald Simon, Guido Sauter, Christian Bernreuther: contributed to conception, design, data collection, data analysis and manuscript writing. Franziska Büscheck, Natalia Gorbokon, Maximilian Lennartz, Andreas M Luebke, Clara von Bargen, Eike Burandt, Andreas H Marx, Ahmed A Bawahab, Doris Hoeflmayer, Patrick Lebok, Frank Jacobsen, Ria Uhlig, Sarah Minner, Till S Clauditz, Stefan Steurer, Anne Menz, David Dum, Till Krech: participated in pathology data analysis and data interpretation. Till Krech, Andreas H Marx: collection of samples. Sebastian D. Rico, Christian Bernreuther: immunohistochemistry analysis. Ronald Simon, Maximilian Lennartz, Claudia Hube‐Magg: data analysis. Sebastian Dwertmann Rico, Ronald Simon, Guido Sauter, Christian Bernreuther: study supervision. All authors agree to be accountable for the content of the work.

## CONFLICT OF INTEREST STATEMENT

The MUC6 antibody clone MSVA‐806R was provided from MS Validated Antibodies GmbH (owned by a family member of GS).

## Supporting information

Supporting information.

Supporting information.
