# Supplementary figures and images for: Pattern of MUC6 expression across 119 different tumor types: A tissue microarray study on 15 412 tumors
Source: Pathol Int. 2023 Apr 14;73(7):281–96. doi: 10.1111/pin.13322 (PMC11551819; doi:10.1111/pin.13322)

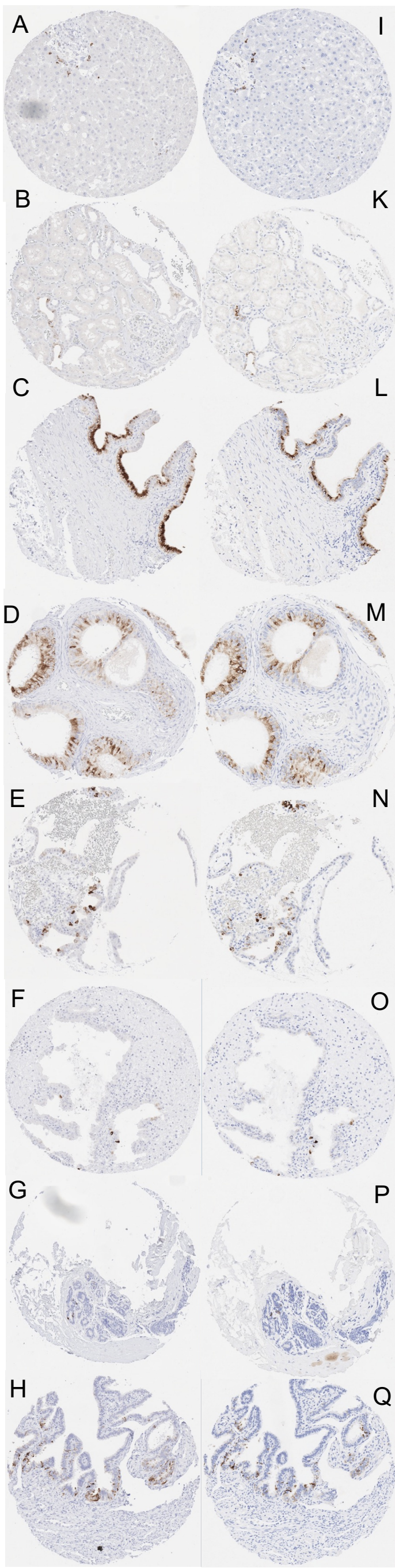

Supplement: Supplementary file 1 — Supporting information. [file PIN-73-281-s002.pdf]
