# Supplementary material for: Pattern of MUC6 expression across 119 different tumor types: A tissue microarray study on 15 412 tumors
Source: Pathol Int. 2023 Apr 14;73(7):281–96. doi: 10.1111/pin.13322 (PMC11551819; doi:10.1111/pin.13322)
Supplement: Supplementary file 2 — Supporting information. [file PIN-73-281-s001.docx]

**Suppl Fig. 1. IHC validation by comparison of antibodies.** The panels show a concordance of immunostaining results obtained by two independent MUC6 antibodies (MSVA-806R, CLH5). Using MSVA-806R, a cytoplasmic positivity was seen in small juxtaportal bile ducts of the liver (A), few collecting ducts of the kidney (B), a subset of epithelial cells of the gallbladder (C), epithelial cells of the epididymis (D), some trophoblastic cells of the early placenta (E), a subset of endometrial cells of the pregnant uterus (F), some luminal epithelial cells of the breast (G), and a subset of epithelial cells of the fallopian tube (H). Using clone CLH5, a staining of identical cell types was seen in the liver (I), kidney (K), gallbladder (L), epididymis (M), placenta (N), endometrium in pregnancy (O), breast (P) and the fallopian tube (Q) The images A-H and I-Q are from consecutive tissue sections.
